# Supplementary material for: The PIK3CA/AKT pathway drives therapy resistance in rhabdomyosarcoma
Source: Nat Commun. 2025 Dec 10;17:65. doi: 10.1038/s41467-025-66632-9 (PMC12770561; doi:10.1038/s41467-025-66632-9)
Supplement: Supplementary file 1 — Supplementary Information [file 41467_2025_66632_MOESM1_ESM.pdf]

Supplemental Information

The PIK3CA/AKT pathway drives therapy resistance in rhabdomyosarcoma

Qiqi Yang[1,2,3,4]**, Yueyang Wang[1,2,3]**, Luis A. Corchete Sanchez[2], Sabateeshan Mathavarajah[1,2,3], Qian Qin[1,2,3,5], Yun Wei[1,2,3], Eric Alpert[1,2,3], Lauren Whelton[1,2,3], Priyanshu Sharma[1,2,3], Stephanie Strom[1,2,3], Ilyas Oultache[1,2,3], A. John Iafrate [1,2], Luca Pinello[1,2,5], Esther Rheinbay[2], Chuan Yan[1,2,3,4]*, David M. Langenau[1,2,3] *

1. Molecular Pathology Unit, Massachusetts General Hospital, Charlestown, MA 02129, USA.
2. Krantz Family Center for Cancer Research, Massachusetts General Hospital, Charlestown, MA 02129, USA.
3. Harvard Stem Cell Institute, Cambridge, MA 02139, USA.
4. Institute of Molecular and Cell Biology, Agency for Science, Technology, and Research (A*STAR), Singapore 138673, Singapore.
5. Broad Institute of MIT and Harvard, Cambridge, MA 02141, USA.

*Co-corresponding Author: David M. Langenau, PhD
Professor of Pathology, Harvard Medical School
Molecular Pathology, Massachusetts General Hospital
149 13th Street, office #6012
Charlestown, Massachusetts 02129, United States of America
dlangenau@mgh.harvard.edu
617-643-6508

Yan Chuan, PhD
Principal Investigator, Institute of Molecular and Cell Biology,
Agency for Science, Technology and Research
61 Biopolis Drive, Proteos, #06-07b
Singapore 138673, Republic of Singapore
yan_chuan@a-star.edu.sg
+65 8727 1957

**Equal contribution

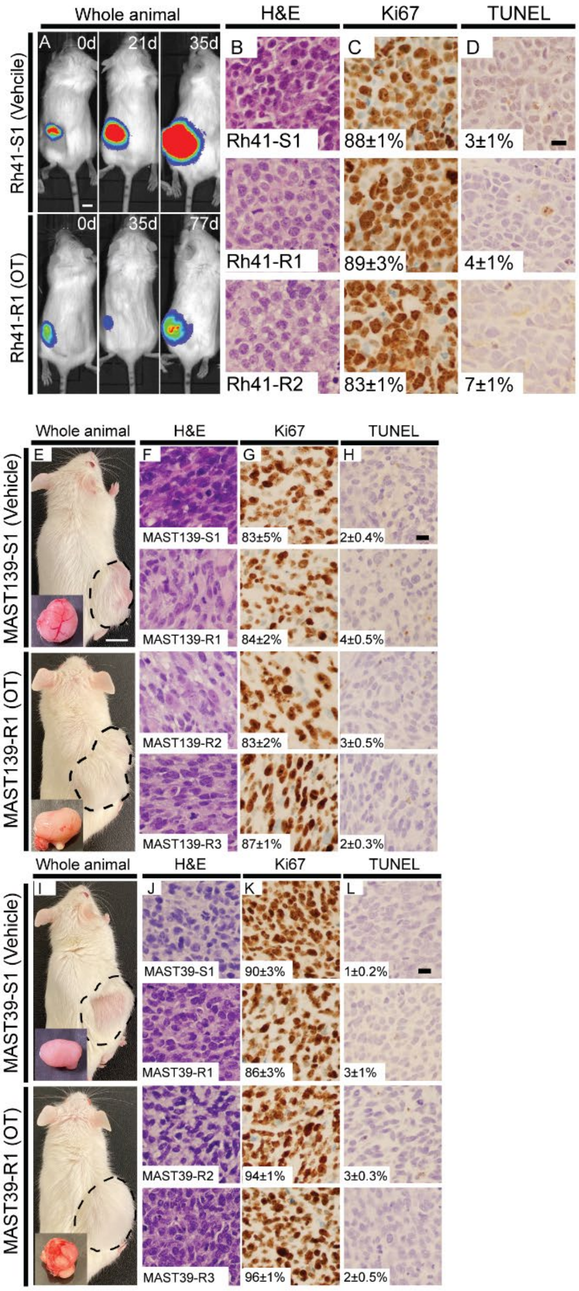

Supplementary Figure 1. Histopathology of xenograft models treated with vehicle or after developing resistance to combination OT therapy. A-D) FP-Rh41, **E-H)** FN-MAST139 PDXs, and **I-L)** FN-MAST39. Bioluminescent imaging completed prior to drug administration (0 days) and after treatment (A). Whole animal images of PDX models (E,I) and images of isolated tumor

(inset, E,I). Representative hematoxylin and eosin-stained sections (B,F,J). IHC for Ki67 (C,G,K) and TUNEL (D,H,L) with average percent positive cells ± standard deviation quantified across three imaging planes noted (n=3 image areas/slide). Scale bar equals 0.5cm (A, E, I), 25 μm (B-D, F-H, J-L).

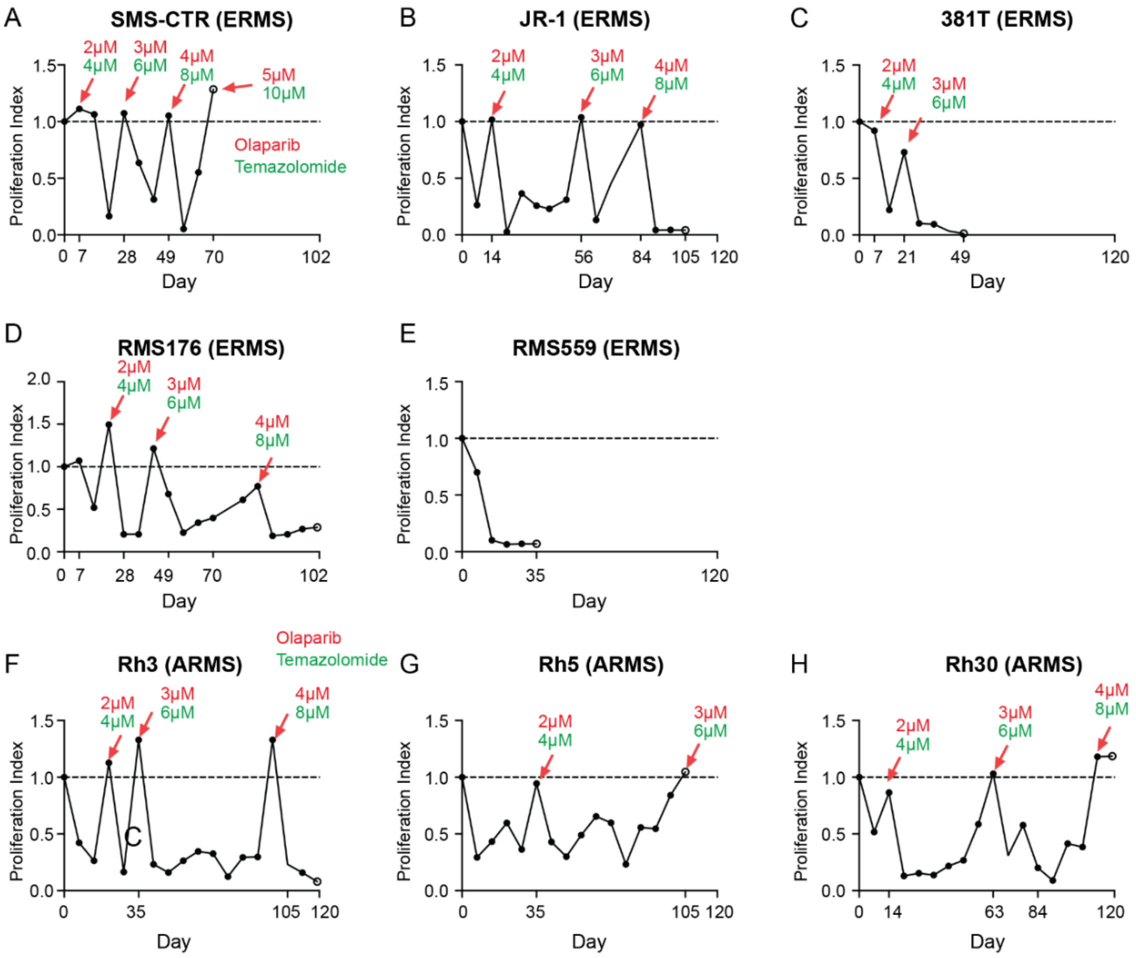

Supplementary Figure 2. Many RMS cell line models become resistant to OT *in vitro.*

Escalating doses of OT were provided to a panel of FN- (A-E: SMS-CTR, JR-1, 381T, RMS176, RMS559) and FP-RMS (F-H: Rh3, Rh5, Rh30). Dose of Olaparib is labeled in red and Tamozolomide concentration is labeled in green. Cells were assessed for relative growth based on manual inspection and reaching confluence (a proliferation index of 1.0). After reaching confluence, cells were re-treated with escalating doses of OT.

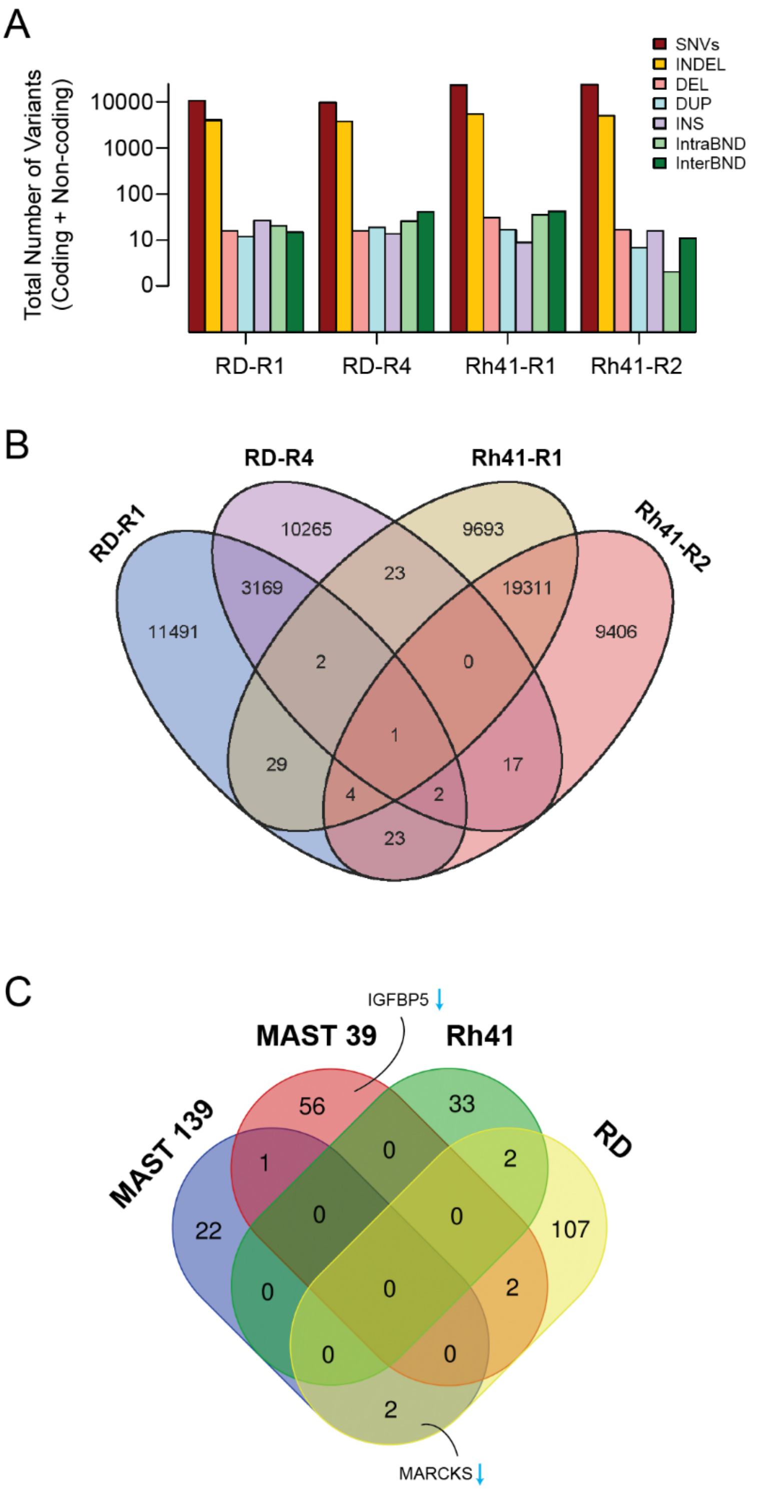

Supplementary Figure 3. Total number of coding and non-coding variants detected in comparing resistant models with parental sensitive models following whole genome

sequencing. A) Graph showing numbers of Single Nucleotide Variants (SNVs), short insertion and deletions (INDEL, <50 nt changes), deletions (DEL, ≥50 nt changes), tandem duplications (DUP, ≥50 nt), insertions (INS, ≥50 nt), intra-chromosomal translocations (intraBND), inter-chromosomal translocations (interBND). ≥ 3 supporting reads in resistant models. No filtering was completed based on allele fraction to capture the maximal number of possible mutations. **B)** Venn Diagram showing acquired SNVs and INDELs in each resistant model. **C)** Venn diagram showing genes down-regulated in comparing parental, sensitive and resistant models in single cell RNA sequencing.

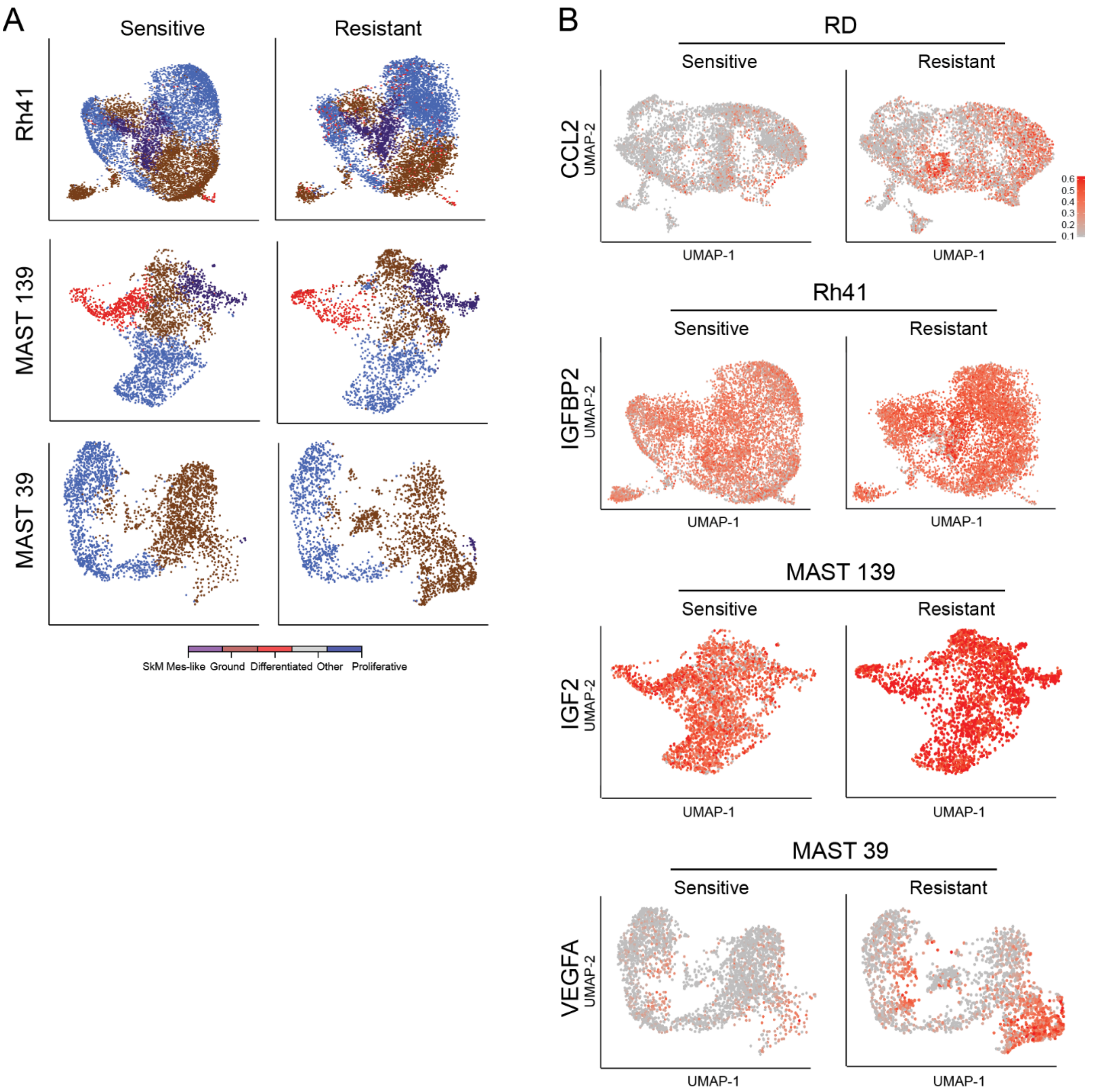

Supplementary Figure 4. TSNE plots denoting RMS cell state (A) and expression of genes of interest from single-cell RNA sequencing experiments (B). Models analyzed included RD-S, RD-R1, Rh41-S, Rh41-R2, MAST39-S, MAST39-R1, MAST139-S, and MAST139-R1.

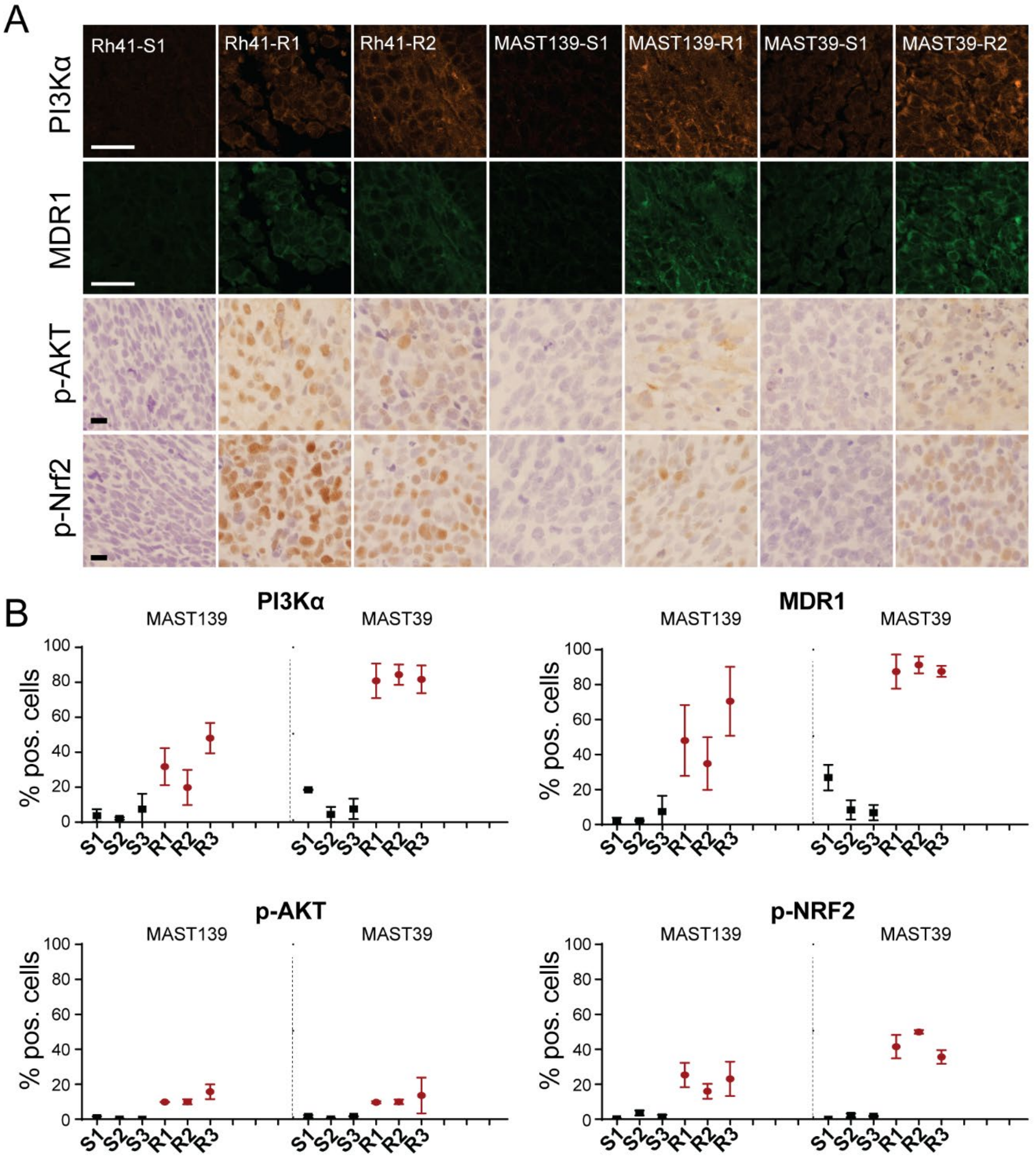

Supplementary Figure 5. The PIK3CA/AKT pathway is upregulated in a large fraction of OT resistant RMS FN and FP models. A) Representative images of IHC staining for p-AKT (Ser473), and p-NRF2 (Ser40) of parental, sensitive Rh41-S1, resistant R1, and R2 (a tumor that continued to respond to therapy after five rounds of OT), sensitive MAST139-S1, resistant R1, and sensitive MAST39-S1, resistant R2. **B)** Quantification of IHC staining for MAST139 and MAST39 (n=3 image areas/slide, data are mean ± SD). Tumors from 3 different mice (biological

replicates) were sectioned, and 3 image areas per tumor (technical replicates) were captured and analyzed. Scale bar equals 25 μm (A).

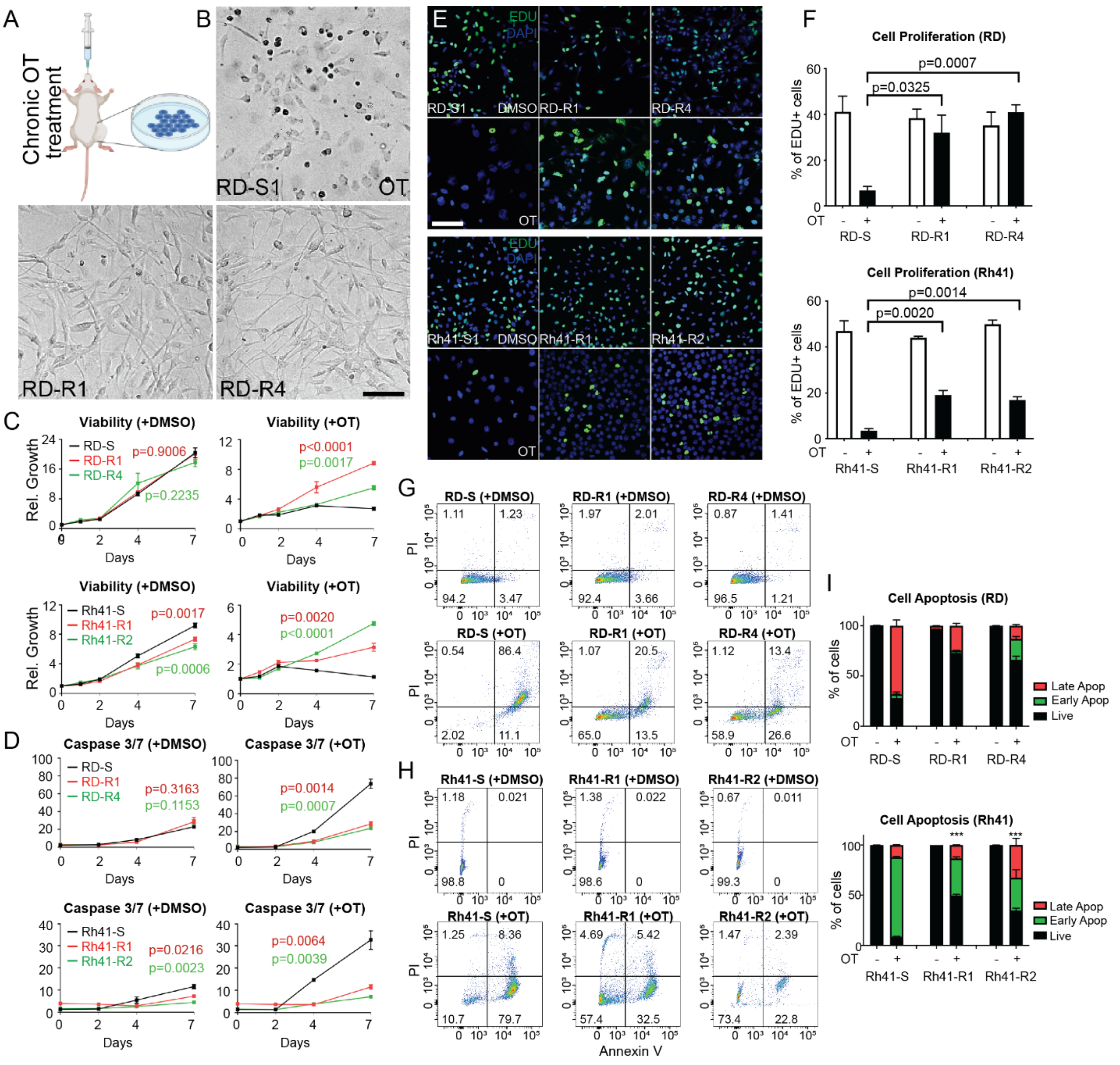

Supplementary Figure 6. Xenograft OT-resistant RD and Rh41 cells retain drug resistance following early passage *in vitro*. A) Schematic of experimental design created in BioRender. Mathavarajah, S. (2025) https://BioRender.com/xqvtar6. **B)** Representative brightfield images

showing RD cell models after treatment with OT for 4 days. Note dead and dying cells in RD-S1 compared with RD-R1 and -R4 models. Similar results seen across three independent biological replicates. **C)** Quantification of relative tumor growth assessed by CellTiter-Glo following treatment for 2, 4, 7 days with DMSO (left) or OT (right). RD (top) and Rh41 (bottom). Mean +/- STD. Representative experiment with each data point comprising $\geq$ three biological replicates. Experiment was independently replicated with similar results. **D)** Quantification of apoptosis detected using Caspase-Glo 3/7 Assay following 2, 4, 7 days of treatment with DMSO (left) or OT (right). RD (top) and Rh41 (bottom). Representative experiment showing mean ± SD from three independent biological replicates. Experiment was independently replicated with similar results. **E)** Representative images of EDU-stained cells treated for 4 days with DMSO control or OT treatment (left, E), EDU (green) and cells counterstained with DAPI to mark nuclei (blue). Representative images from three biological replicates. **F)** Quantification of the percentage of EDU-stained cells. Data are mean ± SD from three biological replicates. **G-H)** Representative flow cytometry analysis showing Annexin V/PI staining after 4 days of treatment with DMSO or OT. RD (G) and Rh41 (H). **I)** Quantification of Annexin V/PI staining. *N*=3 biological repeats. Similar results seen in an independently replicated experiment. ANOVA followed by two-tailed Dunnet's post hoc test was used for statistical analysis to compare differences between sensitive and resistant groups (C-D). ANOVA followed by two-sided Student's T-test comparing OT treated cells with vehicle control cells (F, I). *$p < 0.05$; **$p < 0.01$; ***$p < 0.001$; ***$p < 0.0001$.

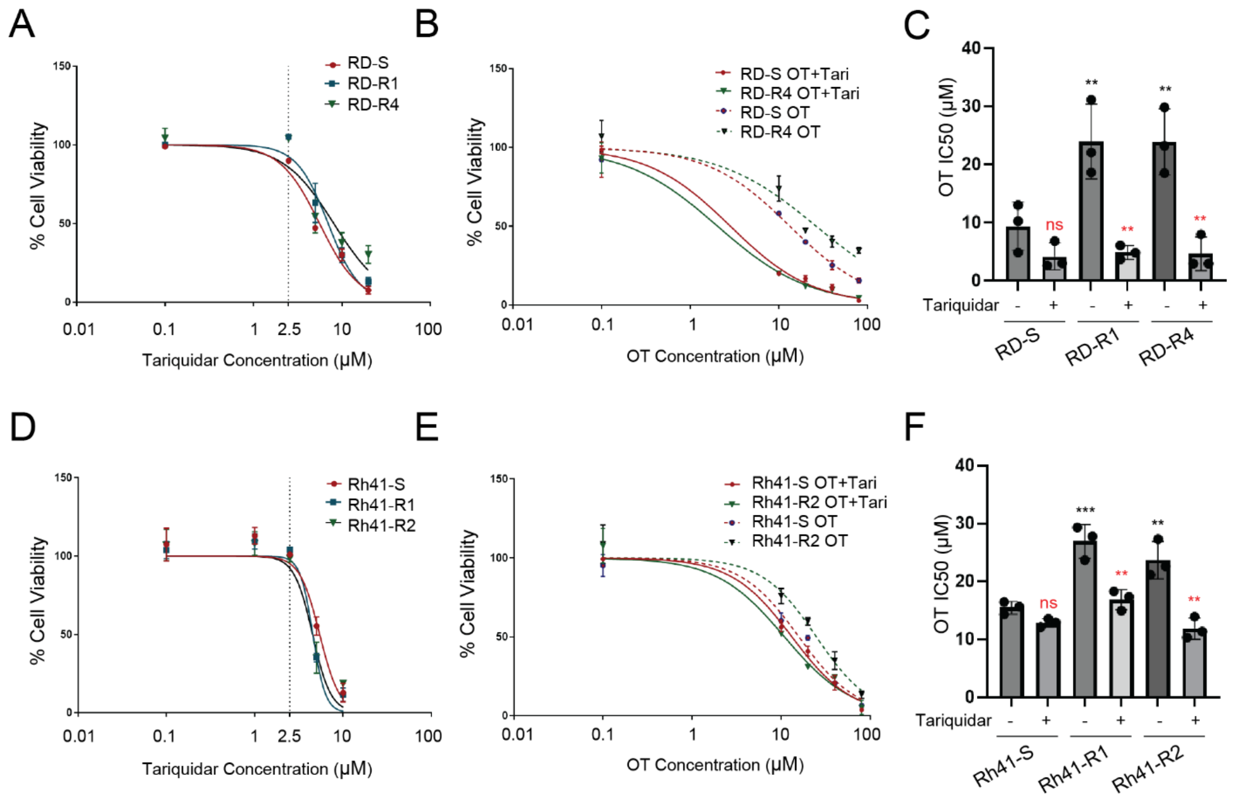

Supplementary Figure 7. Tariquidar ABC transporter inhibitor re-sensitizes resistant RMS clones to OT treatment. A, D) Tariquidar single drug treatment had minimal effects on tumor cell growth when applied to parental or therapy-resistant RD and Rh41 cells (2.5 μM, indicated by dash line). **B-C, E-F)** Tariquidar (dosed at 2.5 μM) re-sensitized resistant RMS to OT induced killing after treatment for 48 hours. Black asterisks denote significance in comparison to the parental sensitive lines (RD-S and Rh41-S for C and F, respectively) by ANOVA followed by Dunnett's post hoc test. Red asterisks denote differences in cell killing by Tariquidar within each model (C and F) by Student's T-test. **p<0.01, ***p<0.001 and not significant (ns). Representative data showing mean ± SD from three biological replicates (A-B, D-E). Similar results were obtained from analysis of two additional independent experiments. Data are mean ± SD from *n*= 3 biological replicates in C and F.

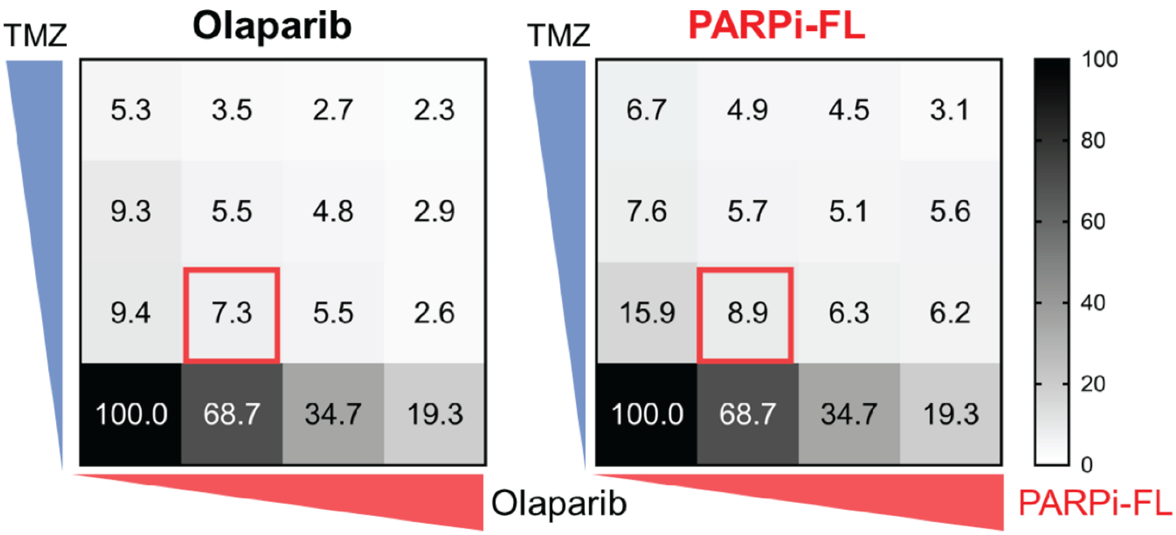

Supplementary Figure 8. Olaparib and PARPi-FL have comparable tumor cell killing when complexed with temozolomide (TMZ). Percent cell viability of RD cells following escalating doses of olaparib + TMZ (left) and PARPi-FL and TMZ (right) treated for 7 days. Dosing for TMZ (0, 10, 20, and 30 μM) and olaparib/PARPi-FL (0, 5, 10, and 15 μM). Red box denotes working concentration used throughout manuscript.

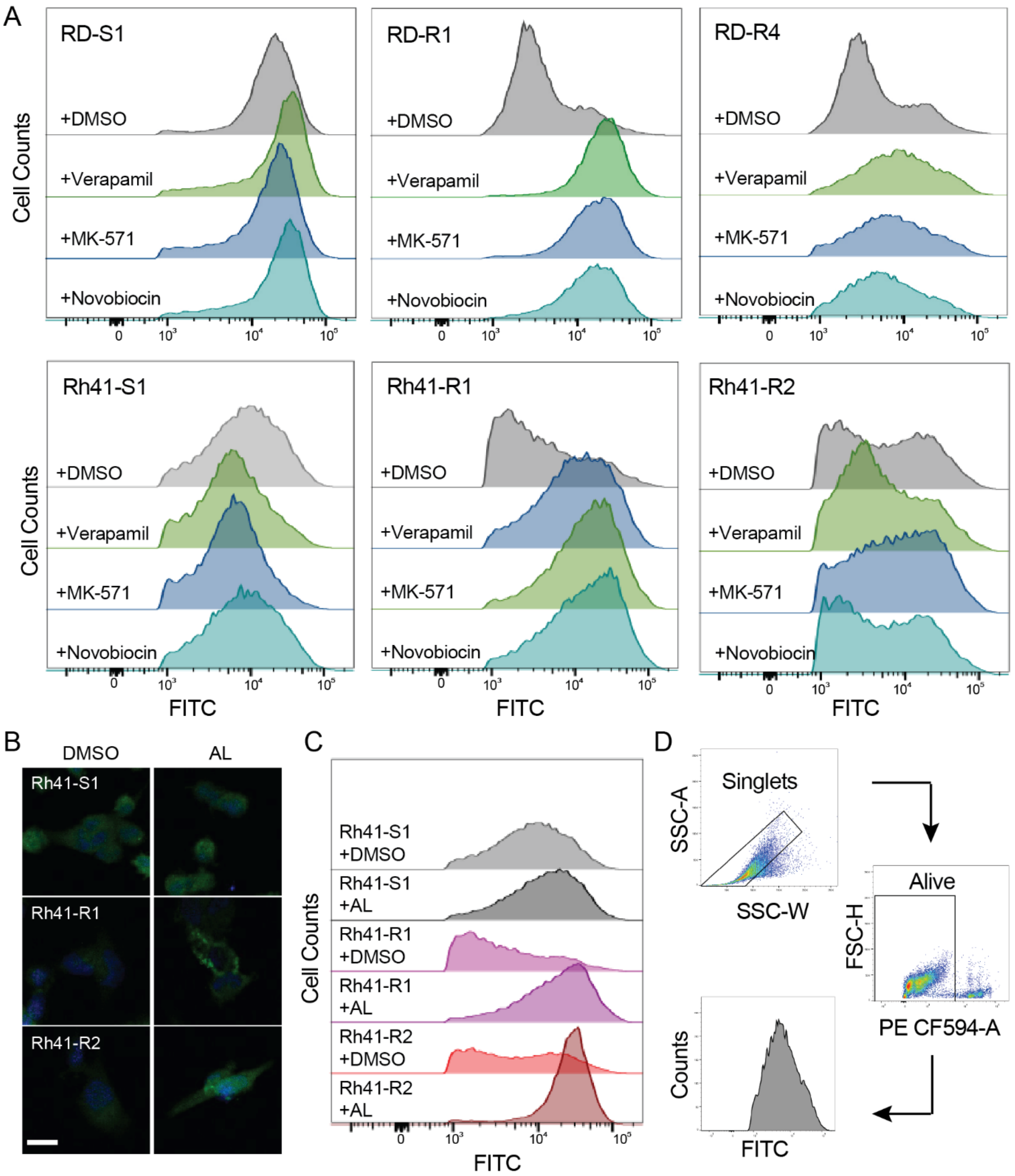

Supplementary Figure 9. ABC drug transporters are more active in resistant RMS cells when assessed using the eFluxx-ID® Green assay. A) Flow cytometric analysis of RMS cells were treated with 0.5% DMSO, 20μM Verapamil (MDR1 inhibitor), 50μM MK-571(MRP inhibitor)

or 50μM Novobiocin (BCRP inhibitor) along with the eFluxx-ID® Green dye for 45 minutes. **B)** Representative images and **C)** flow cytometry analysis of Rh41 parental sensitive (S1) and resistant cells (R1, R2) showing the fluorescence eFluxx-ID® signals after treatment with DMSO or alpelisib PIK3CAα inhibitor (AL). Scale bar equals 10 μm (B). D) The representative FACS gating strategies for analyzing eFluxx-ID® Green dye. Cells were first gated by SSC-A and SSC-W to eliminate non-singlet cells and gated by FSC-H/ PE-CF594-A to eliminate dead cells. (D refers to Figure 3G, Supplementary 9A, C). Data shown are from one representative experiment. Similar results were obtained from an additional independent experiment.

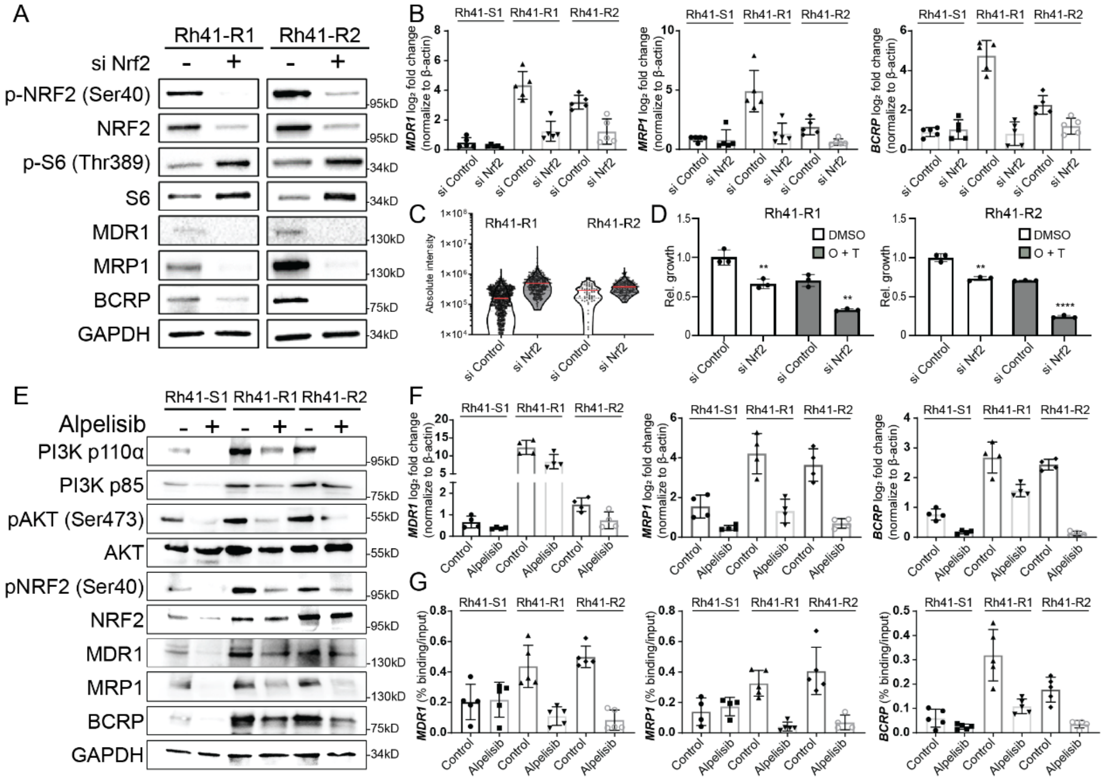

Supplementary Figure 10. NRF2 upregulates ABC transporter expression and function in OT-resistant Rh41 FP-RMS. A-D) siRNA knockdown of NRF2 results in reduced ABC transporter expression and function. **A)** Western blot analysis following 4 days of siRNA knockdown. Data shown are from one representative experiment and was independently verified using additional biological replicates with similar results. The lysates derive from the same experiment but were processed in parallel and run on different gels. One gel was used for analysis of MDR1, Nrf2, p-Nrf2, p-S6, and a second analyzed MRP1, BCRP, and S6. **B)** qRT-PCR showing that ABC transporters are transcriptionally upregulated in resistant clones and can be down regulated following siNRF2. Data shows results for technical replicates, but was independently confirmed three times using additional biological replicates. **C)** Quantification

of PARPi-FL within individual resistant models after 4 days of siRNA knock-down. n≥66 cells per condition. An independent biological replicated experiment showed similar results. **D)** CellTiter-Glo analysis showing relative growth of cells after 7 days of DMSO or OT drug treatment in siControl or siNRF2 knockdown cells. Asterisks denote differences in siControl and siNRF2 in each drug treatment group. An independent biological replicated experiment showed similar results. **E-F)** Alpelisib PIK3Cα inhibitor reduces ABC transporter expression. Western blot (E) or qRT-PCR analysis (F, samples normalized to GAPDH) following 4 days of alpelisib treatment. Western data are from one representative experiment and was independently verified using additional biological replicates with similar results. The lysates derive from the same experiment but were processed in parallel and run on different gels. One gel was used for analysis of PI3K p85, MDR1, MRP1, p-Nrf2, BCRP, and a second for analysis of PI3K p110, Nrf2, AKT, p-AKT. qPCR results were performed three times as independent biological replicates with similar results. **G)** ChIP-qPCR analysis of NRF2 occupancy on the promoters/enhancers of ABCB1 (MDR1), ABCC1 (MRP1) and ABCG2 (BCRP) following treatment with vehicle or alpelisib for 4 days. Data shows mean ± SD from technical replicates and was independently repeated three times with similar results using biological replicates. P-values denote comparison to si Control treated samples using Student's T- test (D). $P < 0.05$ was considered statistically significant.

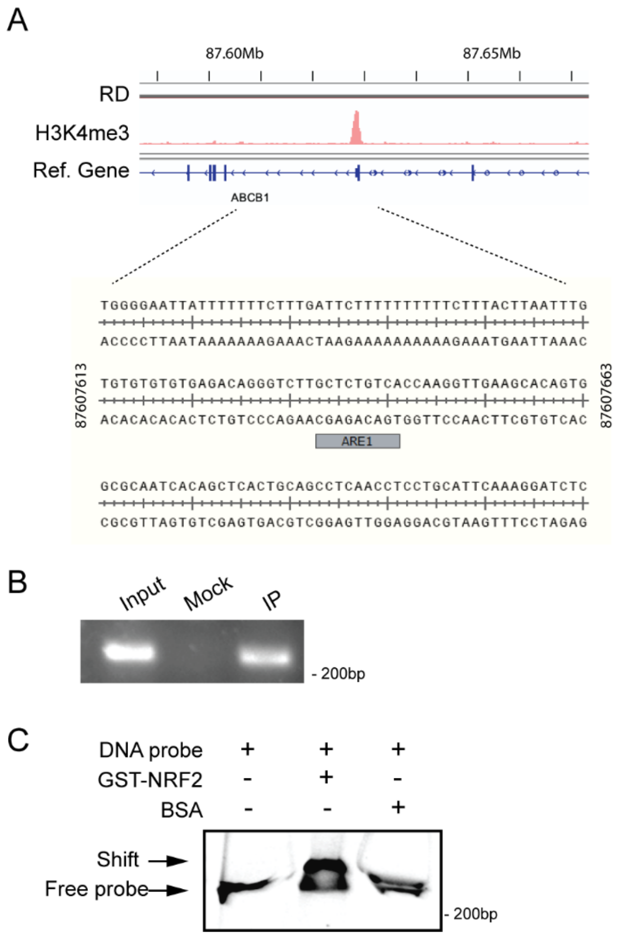

Supplementary Figure 11. NRF2 binds enhancer/promoter elements within human *ABCB1*. A) Genome track displaying ChIPseq of human RD cells with H3K4me3, which marks open chromatin and active transcription near the start site of ABCB1 (top). An ARE was identified in this region using the TFBIND tool (https://tfbind.hgc.jp/, bottom). The result of this sample has been deposited onto the GEO dataset under accession number GSE274640. **B)** NRF2 binds to the ABCB1 site as assessed by Chromatin immunoprecipitation (ChIP) enrichment. PCR was completed using DNA fragments immunoprecipitated using anti-NRF2 in RD cells (IP). Comparison was made between total DNA (Input) and DNA fragments immunoprecipitated by anti-IgG in RD cells (Mock). **C)** Mobility shift assay showing NRF2 binds to DNA containing ABCB1 ARE sequence from RD cells. Data shown in panels B and C were each performed twice independently using two biological replicates with similar results.

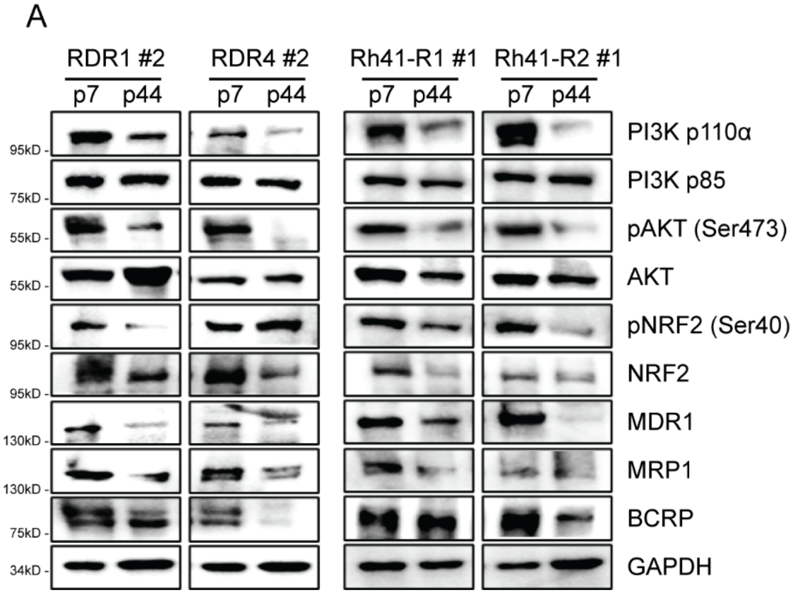

Supplementary Figure 12. Representative Western blots of serial passaged OT-resistant models that re-acquired therapy sensitivity over time. Passage number noted for each model. Each model was derived from a single cell by FACs and cell culture expansion *in vitro.* Western data are from one representative experiment and was independently validated in a second biologically replicated experiment. The lysates derive from the same experiment but were processed in parallel and run on different gels. The gels containing RD samples included one for analysis of PI3K p110, Nrf2, MRP1, and a second for PI3K p85, MDR1, BCRP, p-Nrf2, AKT, p-AKT. The gels containing Rh41 samples included one for analysis of MRP1, Nrf2, p-Nrf2, a second for PI3K p85, MDR1, BCRP, AKT, p-AKT, and a third for PI3K p110.

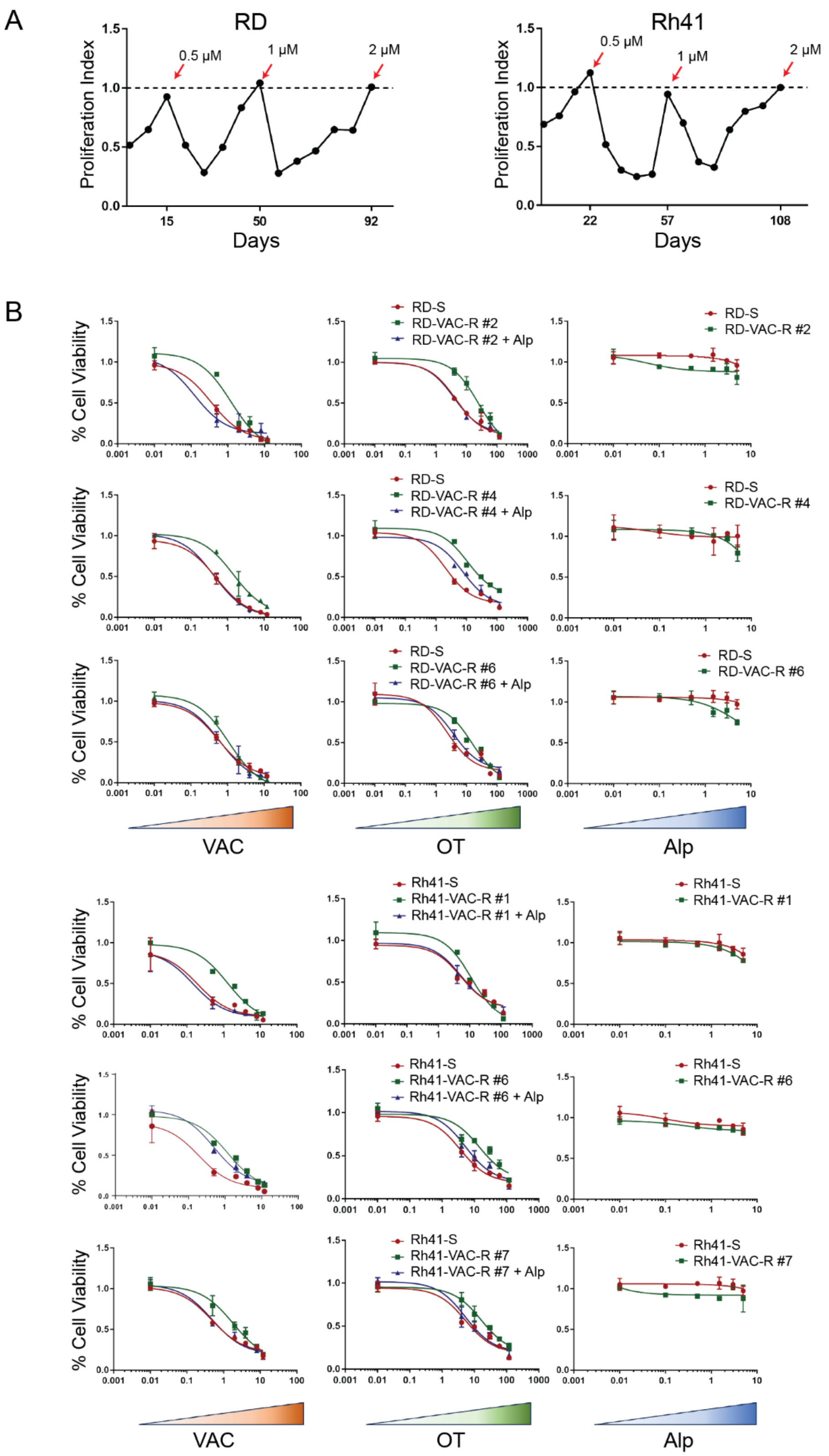
A
RD
Rh41
Proliferation Index
0.5 μM
1 μM
2 μM
Days
B
% Cell Viability
RD-S
RD-VAC-R #2
RD-VAC-R #2 + Alp
RD-VAC-R #4
RD-VAC-R #4 + Alp
RD-VAC-R #6
RD-VAC-R #6 + Alp
VAC
OT
Alp
Rh41-S
Rh41-VAC-R #1
Rh41-VAC-R #1 + Alp
Rh41-VAC-R #6
Rh41-VAC-R #6 + Alp
Rh41-VAC-R #7
Rh41-VAC-R #7 + Alp

Supplementary Figure 13. VAC-resistant RMS can be killed by co-treatment with VAC or OT and alpelisib. A) Development of acquired resistance to VAC in RD and Rh41 cells. Following passage of cells in escalating doses of VAC, individual clones were expanded from single cells after FACS into 96-well plates. **B)** Alpelisib re-sensitized VAC resistant RMS to VAC or OT induced killing after treatment for 48 hours. Alpelsib single drug treatment had minimal effects on tumor cell growth when applied (4 μM). Representative experiment showing results from three independent biological replicates. This experiment was performed three times using biological replicates, yielding similar results across all experiments.
