# Supplementary material for: The PIK3CA/AKT pathway drives therapy resistance in rhabdomyosarcoma
Source: Nat Commun. 2025 Dec 10;17:65. doi: 10.1038/s41467-025-66632-9 (PMC12770561; doi:10.1038/s41467-025-66632-9)
Supplement: Supplementary file 2 — Description of Addtional Supplementary Files [file 41467_2025_66632_MOESM2_ESM.pdf]

Description of Additional Supplementary Files

Supplementary Data 1. Summary of genomic mutations found in parental and OT resistant RMS models analyzed by clinical grade SNaPshot analysis. Changes with >0.2 allele frequency shown (allele freq.). Copy number ratio (CNR). Not detected (ND). Red text denotes changes between parental and resistant tumors.

Supplementary Data 2. Genomic DNA alterations identified by whole genome sequencing comparing therapy sensitive and OT resistant RMS models. Exonic and splicing single nucleotide variants (SNVs) and short exonic deletions noted. >20 reads/resistance sample and variant allele fraction of >0.3 was used as cut off. SNVs and INDELS reported based on being <1% represented in the gnomad database for non-Finish European populations. Data used to generate Venn diagram shown in Figure 2B.

Supplementary Data 3. Analysis of PIK3 pathway regulators assessed by whole genome sequencing with expected impact on pathway deregulation noted. Events with variant allele fraction >30% are reported as candidate drivers. Not applicable (NA).

Supplementary Data 4. IC50 values and PIK3CA expression in resistant clones that revert to therapy sensitive states after serial passaging in that absence of therapy. Data used to generate bar graphs shown in Figure 2E.

Supplementary Data 5. Analysis of 13 small molecule inhibitors for their ability to kill parental and resistant RD and Rh41 tumors alone or in combination with OT. Not done (ND). Data used to generate heat maps shown in Figure 3C in main text.

Supplementary Data 6. Quantification of PI3K/AKT/ABC transporters protein levels in PI3K H1047R and myr-AKT overexpressing RMS cells.

Supplementary Data 7. PCR primers.

Supplementary Data 8. RNA expression alterations identified by single cell RNA sequencing comparing therapy sensitive and OT resistant RMS models. Data used to generate Venn diagram shown in Figure 2G and Supplementary Figure 3C.
