# Supplementary material for: The PIK3CA/AKT pathway drives therapy resistance in rhabdomyosarcoma
Source: Nat Commun. 2025 Dec 10;17:65. doi: 10.1038/s41467-025-66632-9 (PMC12770561; doi:10.1038/s41467-025-66632-9)
Supplement: Supplementary file 11 — Reporting Summary [file 41467_2025_66632_MOESM11_ESM.pdf]

nature portfolio

Corresponding author(s): David M. Langenau, Chuan Yan

Last updated by author(s): 7.9.2025

Reporting Summary

Nature Portfolio wishes to improve the reproducibility of the work that we publish. This form provides structure for consistency and transparency in reporting. For further information on Nature Portfolio policies, see our Editorial Policies and the Editorial Policy Checklist.

Statistics

For all statistical analyses, confirm that the following items are present in the figure legend, table legend, main text, or Methods section.

n/a	Confirmed	
☐	☒	The exact sample size (*n*) for each experimental group/condition, given as a discrete number and unit of measurement
☐	☒	A statement on whether measurements were taken from distinct samples or whether the same sample was measured repeatedly
☐	☒	The statistical test(s) used AND whether they are one- or two-sided<br>*Only common tests should be described solely by name; describe more complex techniques in the Methods section.*
☐	☒	A description of all covariates tested
☐	☒	A description of any assumptions or corrections, such as tests of normality and adjustment for multiple comparisons
☐	☒	A full description of the statistical parameters including central tendency (e.g. means) or other basic estimates (e.g. regression coefficient) AND variation (e.g. standard deviation) or associated estimates of uncertainty (e.g. confidence intervals)
☐	☒	For null hypothesis testing, the test statistic (e.g. *F*, *t*, *r*) with confidence intervals, effect sizes, degrees of freedom and *P* value noted<br>*Give P values as exact values whenever suitable.*
☒	☐	For Bayesian analysis, information on the choice of priors and Markov chain Monte Carlo settings
☒	☐	For hierarchical and complex designs, identification of the appropriate level for tests and full reporting of outcomes
☒	☐	Estimates of effect sizes (e.g. Cohen's *d*, Pearson's *r*), indicating how they were calculated

Our web collection on statistics for biologists contains articles on many of the points above.

Software and code

Policy information about availability of computer code

Data collection	Single cell sequencing data were demultiplexed and processed using the 10X cellranger 3.1.0 pipeline to generate the read count matrices for both resistant and sensitive clones. Xenograft whole -genome sequencing was performed using the NovaSeq X Plus sequencer (Novogene) to obtain 150 base pair (bp) paired reads. Read quality statistics were obtained with FastQC (v.0.12.1) ("Babraham Bioinformatics - FastQC A Quality Control tool for High Throughput Sequence Data," n.d.).
Data analysis	These codes for single cell sequencing have been uploaded to GitHub repo (https://github.com/qinqian/rms_analysis/tree/master/drug). Detailed description of whole genome sequencing analysis is included in supplementary information.

For manuscripts utilizing custom algorithms or software that are central to the research but not yet described in published literature, software must be made available to editors and reviewers. We strongly encourage code deposition in a community repository (e.g. GitHub). See the Nature Portfolio guidelines for submitting code & software for further information.

Data

Policy information about availability of data

All manuscripts must include a data availability statement. This statement should provide the following information, where applicable:

- Accession codes, unique identifiers, or web links for publicly available datasets
- A description of any restrictions on data availability
- For clinical datasets or third party data, please ensure that the statement adheres to our policy

The scRNA-seq data generated from mouse tumor samples in this study have been deposited in the Gene Expression Omnibus (GEO), a public functional genomics data repository, under the accession number GSE280546. The database for Supplementary Figure 11A is under the accession number GSE274640 (GEO). The Whole Genome Sequencing data generated from the drug-sensitive and resistant samples have been deposited in the Sequence Read Archive (SRA) database, under the accession number PRJNA1061089. All data are included in the Supplementary Information or available from the authors, as are unique reagents used in this Article. The raw numbers for charts and graphs are available in the Source Data file whenever possible. Source data are provided with this paper.

Research involving human participants, their data, or biological material

Policy information about studies with human participants or human data. See also policy information about sex, gender (identity/presentation), and sexual orientation and race, ethnicity and racism.

Reporting on sex and gender	This study did not involve human participants
Reporting on race, ethnicity, or other socially relevant groupings	NA
Population characteristics	NA
Recruitment	NA
Ethics oversight	NA

Note that full information on the approval of the study protocol must also be provided in the manuscript.

Field-specific reporting

Please select the one below that is the best fit for your research. If you are not sure, read the appropriate sections before making your selection.

☒ Life sciences ☐ Behavioural & social sciences ☐ Ecological, evolutionary & environmental sciences

For a reference copy of the document with all sections, see nature.com/documents/nr-reporting-summary-flat.pdf

Life sciences study design

All studies must disclose on these points even when the disclosure is negative.

Sample size	For drug response experiments, each treatment has three replicates and student t test was used to compare the significant difference between each group. Mean plus/minus SEM was used to present the sample variation. In mice xenograft study, 6 mice per aim was used to start the experiments. The sample size was chosen based on the reference paper.
Data exclusions	No data were excluded from the analyses.
Replication	Major findings were repeated two to three times separately as noted in the manuscript.
Randomization	Samples were collected and evenly distributed into subgroups. Treatments were assigned randomly into each subgroup.
Blinding	A subset of data were blinded to the investigators during data quantification as noted in the manuscript.

Reporting for specific materials, systems and methods

We require information from authors about some types of materials, experimental systems and methods used in many studies. Here, indicate whether each material, system or method listed is relevant to your study. If you are not sure if a list item applies to your research, read the appropriate section before selecting a response.

Materials & experimental systems

n/a	Involved in the study	
☐	☒	Antibodies
☐	☒	Eukaryotic cell lines
☒	☐	Palaeontology and archaeology
☐	☒	Animals and other organisms
☒	☐	Clinical data
☒	☐	Dual use research of concern
☒	☐	Plants

Methods

n/a	Involved in the study	
☒	☐	ChIP-seq
☐	☒	Flow cytometry
☒	☐	MRI-based neuroimaging

Antibodies

Antibodies used

Primary antibodies used are PTEN (CST, 9559), p-PTEN (Ser380/Thr382/383) (CST, 9551), EGFR (abeam, ab52894), PI3K p85 (CST, 4292), p-PI3K p85 (Tyr458) (CST, 17466), AKT (CST, 4691), p-AKT (Ser473) (CST, 4060), NRF2 (abeam, ab62352), p-NRF2 (Ser40) (abeam, ab76026), 56 (CST, 2317), p-56 (Ser235/236) (CST, 81736), 4EBP1 (CST, 9644), p-4EBP1 (Thr37/46) (CST, 2855), MDRl/ABCBl (CST, 13342), MRPl/ABCCl (CST, 72202), BCRP/ABCG2 (CST, 42078), GAPDH (CST, 5174). Secondary antibodies used included anti-mouse lgG, HRP-linked Antibody (CST, 7076), anti-rabbit lgG, HRP-linked Antibody (CST, 7074).

Validation

All the antibodies were validated by each company and the validation statements can be found on the websites.

Eukaryotic cell lines

Policy information about cell lines and Sex and Gender in Research

Cell line source(s)

Cells lines used in the work are RD, Rh41, SMS-CTR, JR-1, RMS176, 381T, RMS559, Rh30, Rh5 and Rh3. PDX derived cell lines in the study are MAST139, MAST39, MAST118, MSK82489. Cell lines are from ATCC. All PDX derived cell lines are acquired from St. Jude childhood solid tumor network and MSKCC.

Authentication

All human cell lines and PDXs used in this work were authenticated prior to experiments by small tandem repeat profiling using the Whatman Flinders Technology Associates sample collection kit (ATCC)

Mycoplasma contamination

All cell lines are tested negative for mycoplasma contamination from VRL Diagnostics

Commonly misidentified lines
(See ICLAC register)

NA

Animals and other research organisms

Policy information about studies involving animals; ARRIVE guidelines recommended for reporting animal research, and Sex and Gender in Research

Laboratory animals

Female NOD.Cg-Prkdcscid Il2rgtm1Wjl/SzJ (NSG) mice used in this work were grown at Charles River Laboratories.

Wild animals

This study did not involve wild animals.

Reporting on sex

All the mice used in this study are female. Gender effects from the mice were not considered in this study. In total, 120 female mice were used in this study.

Field-collected samples

This study did not contain field-collected samples.

Ethics oversight

All the mice study were guided by Charles River Laboratories.

Note that full information on the approval of the study protocol must also be provided in the manuscript.

Plants

Seed stocks	This study did not contain seed stocks.
Novel plant genotypes	NA
Authentication	NA

Flow Cytometry

Plots

Confirm that:

☒ The axis labels state the marker and fluorochrome used (e.g. CD4-FITC).

☒ The axis scales are clearly visible. Include numbers along axes only for bottom left plot of group (a 'group' is an analysis of identical markers).

☒ All plots are contour plots with outliers or pseudocolor plots.

☒ A numerical value for number of cells or percentage (with statistics) is provided.

Methodology

Sample preparation	Rhabdomyosarcoma cells were incubated with Green detection reagent according to the manufacturer's protocol for 45 min in 37°C. Cells were imaged using confocal microscopy (Zeiss LSM710 inverted microscope) with 20X objective employing 488nm laser or suspended in cold PBS for flow cytometry analysis.
Instrument	The BD LSRFortessa Cell Analyzer was used for the flow cytometry experiment.
Software	FlowJo was used for the flow cytometry analysis.
Cell population abundance	A non-stained control was used as negative control. The cell population abundance was shown by comparing the sensitive and resistant cell samples using the same setting.
Gating strategy	A non-stained control was used for FSC/SSC gating.

☒ Tick this box to confirm that a figure exemplifying the gating strategy is provided in the Supplementary Information.
